# Bibliographical

**Published:** 1872-11

**Authors:** 


### BIBLIOGRAPHICAL.

General and Differential Diagnosis of Ovarian Tumors, with Special Reference to the Operation of Ovariotomy, and Occasional Pathological and Therapeutical Considerations. By Washington L. Atlee, M. D. With thirty-nine illustrations. Philadelphia: J. B. Lippincott & Co., 1873. Through Phillips & Crew, Atlanta, Georgia—through whom orders may be sent.

Ovariotomy, truly, has "taken a position among the established operations of surgery," and among those who have contributed to thus "establish" it, none rank higher than the author of the treatise before us. "The book," he writes, "is a mere transcript of his own clinical experience, recorded at the moment of examination, and comprises the result of observations continuously pursued since the year 1843." The author gives the general diagnosis of ovarian tumors, with cases: ascites; peritoneal cysts, or cysts of the broad ligament; other cystic tumors of the abdomen; dermoid tumors; pregnancy; pregnancy coexisting with ovarian dropsy; tumors of the uterus; retroversion of the uterus; retention of the menstrual fluid; dropsy of the amnion; enlargement of the adbominal viscera; distention of the urinary bladder; accumulation of fæces in the intestines; accumulation of gas in the intestines; inflammatory deposits in the pelvic and abdominal cavities; pelvic tumor and abscess; spinal curvature, lumbar or psoas abscess; malignant tumors of the abdomen; rectocele and cystocele; hypertrophy of the abdominal wall; adhesions; pathology of cystic tumors of the ovary; dropsical fluids of the abdomen; their physical properties; chemical analysis; microscopic appearance of and diagnostic value; based on the examination of several hundred specimens.

The book will be highly prized by surgeons and physicians generally, and we are pleased to know that the author intends to follow this by a second volume, comprising cases and their management.

Evolution of Life. By Henry C. Chapman, M. D., Member of the Academy of Natural Sciences, Philadelphia. J. B. Lippincott & Co.: 1873. Through Phillips & Crew, Atlanta, Ga.

The design of the author is to bring together a condensed view of the evidences for the theory that the animal and vegetal worlds have been very gradually developed or evolved, as distinguished from the hypothesis of their sudden, special creation. In attempting to do this, the author places before the reader "the most important generalizations in reference to the structure of plants and animals, their petrified remains, and mode of development, and attempts to point out how the theory of the Evolution of Life follows from the acts of anatomy, geology and embryology." In other words, the author, in as popular manner as possible, attempts to support and defend the views of Mr. Darwin on the Origin of the Species.

The doctrine of Evolution, as we understand it, is founded upon a theoretical tripod: *First*, the nebula hypothesis of La Place, which accounts for the formation of worlds and systems of worlds from nebulæ, or fire-mist, the gradual condensation of which, in our planetary system, constituted the sun, from which, in some eruptive convulsion, the various planets were thrown off into space, and around which they have ever since revolved, losing their heat and becoming condensed. At some remote period after this condensation, vegetable life spontaneously sprung into existence, and from which, in succeeding ages, animal life was evolved. So we have the *second hypothesis*—the doctrine of spontaneous generation—a theory to show the natural origin of life from the simpler kinds of organisms; and, *third*, the doctrine of the transmutation of species, or the gradual evolution of a few simple species spontaneously produced, from which, by "the survival of the fittest," or by "natural selection," the higher order of plants and animals sprung.

To say we believed all this, would be equivalent to saying we rejected the Mosaic record of creation. The adherents of the evolution theory of life would have us accept their theories at no less a sacrifice—a sacrifice which, in its completeness, would rob the world of a Divine Revelation, and the Christian of his Saviour.

The doctrine of the Evolution of Life is not only opposed to Revelation, but to Science—for it is only a speculation, which the mass of scientific men reject. Among these, we call to mind Agassiz, Miller, Sedgewick, Brewster, and the younger Herschell.

"So-called" Science, in its efforts to overthrow Revelation, is a most dangerous and treacherous guide. How shall we judge it? By its past. The facts of science we receive, but we utterly reject the theories built upon them. We ask, have scientists never embraced the emptiest speculations? Have they not been, again and again, forced to disown theories for which they fought and urged as true? Has there been no wrangling among the philosophers of the world? When man attempts to study man—man who can be handled, during life and after death—is he always correct in his interpretations of the facts he discovers? Let the exploded theories of physiologists answer; let the theories of practice, which have been as various and conflicting as possible, answer! If the most sagacious blunder in the investigations of himself, how shall he succeed in unfolding to man the most difficult of all studies—aside from the Mosaic record—that of his origin?

We are told that Mathematics is an exact science, and yet mathematicians have ridden their hobbies with their differentials and integrals! If it is so with Mathematics, shall we be surprised that Geology, the veriest infant among the sciences, should be tortured by the silly speculations of its votaries? True science has ever borne testimony to the truth of Revelation, and, although the scientist and the theologian have traveled different roads, they both meet, at last, at the Source of all Truth—in God.

In opposition to the evolution theory of life, Agassiz writes: "I wish to enter my earnest protest against the transmutation theory. It is my belief that naturalists are chasing a phantom in their search after some material gradation among created beings, by which the whole animal kingdom may have been derived by successive development from a single germ, or from a few germs. I confess that there seems to me a repulsive poverty in this material explanation that is contradicted by the intellectual grandeur of the Universe. I insist that this theory is opposed to the processes of Nature, as we have been able to apprehend them."

The work of Dr. Chapman contains a multitude of facts—facts upon which he attempts to support his belief of the evolution of life. When however, he departs from his facts, and plunges into the mists of speculation, we pause in our admiration of the facts to lament over his folly. As an example of this, we will exhibit one trunk of his "seventh tree." Looking for our ancestry, he points us, first, to the monotremata; then to marsupials; opossums; half-apes; lemur; monkeys; simiæ (man-like monkeys); extinct money-like man; man.

As before remarked, the work is replete with facts, and, however much we do condemn the evolution theory, yet it will afford the reader much wholesome instruction, and an insight into one of the flightiest and wildest speculations of the age. The book is handsomely bound, and the letter-press is unsurpassed.

The Ten Laws of Health; or, How Disease is Produced and Can be Prevented. By J. R. Black, M. D. Philadelphia: J. B. Lippincott & Co., 1872.

This is a very readable and instructive work, designed for both the physician and the non-professional reader. The ten laws laid down—rather arbitrarily, many may think—are fully discussed, and inculcate wholesome hygienic rules, the observance of which, by the people, would be of the greatest importance. The physician will find in them many valuable suggestions, which he can employ for the good of his constituency. The ten laws discussed by the author are as follows: Breathing of pure air—violation and results; adequate and wholesome food and drink—violation and results; adequate out-door exercise—violation and results; adequate and unconstraining covering for the body—violation and results; the exercise of the sexual function only for, and no interference with, the natural course of reproduction; a habitation in the climate for which the constitution of the body is adapted; pursuits which do not cramp or overtask any part of the body, or subject it to irritable and poisonous substances; personal cleanliness; tranquil states of the mind, and adequate rest and sleep; no intermarriage of near blood relations. To each of the laws the author devotes a chapter, and "on the mode of observing" each law a chapter. It is well written, and the work finely printed.

Practical Lessons in the Nature and Treatment of the Affections Produced by the Contagious Diseases, with an Account of the Primary Syphilitic Poison, and of its Communicability, based on Extensive, Direct and Comparative Observation of the Diseases in both Sexes; with an Appendix on the Recent Report of the Royal Commission on how the Contagious Diseases act, and its Application to the Voluntary Hospital System. By John Morgan, A. M., M. D., University of Dublin. With plain and colored Illustrations. Philadelphia: J. B. Lippincott & Co., 1872.

The author has furnished an exceedingly interesting work, of "moderate dimensions, illustrated, and of a reasonable price." In fact, he has placed an excellent work on the "Contagious Diseases" within the reach of the humblest practitioner. Want of space forbids an elaborate notice, yet we feel bound to say that the author has "shown that disease can be produced from the contact and admission of a secretion unconnected with any local sore, but dependent on constitutional taint."

The work is handsomely illustrated, and should be in the hands of all practitioners, being, to our thinking, the very best work, for them, before the profession.

Hand-Book of Compound Medicines; or, the Prescriber's and Dispenser's Vade-Mecum. By Arnold J. Cooley. Philadelphia: J. B. Lippincott & Co., 1873.

This is a work of 219 pages, and one of the most valuable and comprehensive of its class. Printed in small type and compact, it presents a mass of information nowhere else to be found in one volume. The work is divided into two parts. Part I contains: Pills, boluses, globules, grains and granules, pharmacopœal, hospital and magistral, their preparations, formulæ, doses, leading uses and synonyms, including quack medicines. Part II contains: Mixtures, pharmacopœal, hospital and magistral, their preparation, formulæ, doses, leading uses and synonyms, including quack medicines.

The Transactions of the American Medical Association. Volume XXIII. Philadelphia: Printed for the Association.

This is a fine volume, and one we think the Association will be proud of. We will not attempt an extended notice, as want of space preculdes our doing the volume justice. We hope our readers will procure a copy and read it for themselves. The Permanent Secretary, Dr. W. B. Atkinson, deserves the thanks of the Association and the profession for the handsome manner in which the volume is gotten up. A more genial and excellent gentleman is difficult to find. Those desiring copies will address him at Philadelphia.

